# Developing and implementing a public health co-research program for Special Olympics athletes

**DOI:** 10.1186/s40900-023-00450-5

**Published:** 2023-06-19

**Authors:** Anna-Mariya Kirova, Tyler Jakab, Graham Bartsch, Stephanie Corazzini, Alexis Sokoloff, Erin Delahunty, Rachel Seymour, Eric Rubenstein

**Affiliations:** 1 School of Public Health, Boston University, Boston, MA USA; 2 Special Olympics Massachusetts, Marlborough, MA USA

**Keywords:** Co-research, Public health, Special Olympics, Intellectual and developmental disability

## Abstract

Individuals with intellectual and developmental disabilities are often the subject of research while rarely being included in formulating research questions, planning studies, and making decisions related to protocols and analyses. In turn, most research regarding people with intellectual and developmental disabilities is not carried out by researchers with disabilities themselves. We developed a co-research training program where individuals with intellectual and developmental disabilities were taught about research. The program was designed using best practices and existing materials resources. We recruited four participants from Special Olympics Massachusetts and conducted the training in the Fall of 2021. We evaluated the program with surveys, qualitative interviews, and tracking continued involvement of co-researchers in research projects. Participants were partners in the evaluation process. The training program was six sessions and included lessons about why research is important, how to conduct research, and an experiential learning project where co-researchers conducted a study of their coaches. The program was well received by participants, and one year later they were still involved with research projects. A co-researcher training focused on public health for Special Olympics athletes is feasible and beneficial for athletes, researchers, and Special Olympics programs. However, there are still barriers like a lack of funding and time, that need to be addressed to ensure wide program success.

**Supplementary Information:**

The online version contains supplementary material available at 10.1186/s40900-023-00450-5.

## Abstract (plain language summary)

Co-research offers people with intellectual and developmental disabilities an opportunity to conduct research, rather than be the subjects. We developed a six-session co-researcher training in 2021 focused on public health for Special Olympics athletes. We found that co-research is feasible and rewarding for people with intellectual and developmental disabilities. Additional co-research activities and evaluation are needed. Special Olympics already conducts public health research activities and co-research training could be incorporated.

**Supplementary Information:**

The online version contains supplementary material available at 10.1186/s40900-023-00450-5.

Individuals with intellectual and developmental disabilities are often the subjects of research while rarely being included in formulating research questions, planning studies, and making decisions related to protocols and analyses. In turn, most research regarding people with intellectual and developmental disabilities is not carried out by researchers with disabilities themselves [4, 5]. The lack of inclusion of individuals with intellectual and developmental disabilities on research teams is not only an equity issue, but also hampers scientific advancement. Individuals with intellectual and developmental disabilities are deeply knowledgably about their lived experiences and have training and expertise in many areas relevant to research and policy [5].

Some researchers have aimed to address the lack of inclusion of individuals with intellectual and developmental disabilities in research teams by incorporating those with intellectual and developmental disabilities as advisors throughout the process [3]. While having advisors with intellectual and developmental disabilities is a step forward, the advisory approach still relegates individuals with intellectual and developmental disabilities as separate from the research team and can fall short of complete collaboration or contribution. Co-research is a means of conducting research that fosters complete collaboration between researchers and the communities at the center of research interests [5, 14]. Training individuals with intellectual and developmental disabilities to conduct research themselves as part of existing research teams can better establish them as complete stakeholders and challenge notions of “tokensim,” or when members of underrepresented groups are only given symbolic representation or leadership [2].

Although training individuals with intellectual and developmental disabilities as co-researchers can help overcome equity issues in research, there are barriers that prevent widespread adoption. Namely, individuals with intellectual and developmental disabilities have varying abilities, strengths, and weaknesses, which may complicate necessary and appropriate accommodations for all team members involved [3, 13]. Payment and compensation are a significant concern and hurdle for many potential co-researchers with intellectual and developmental disabilities. In the US, millions of individuals with disabilities receive Supplementary Security Income (SSI); however, eligibility to receive this monthly benefit can be revoked if one earns more than $1767 per month, although individuals are often deemed ineligible with much less earned income when other unearned income and assets are considered by the Social Security Administration [10]. This in turn can lead to a loss of Medicaid covered health insurance. Therefore, if co-researchers with intellectual and developmental disabilities are appropriately financially compensated for their contributions, those who receive SSI may be jeopardizing their eligibility to continue receiving benefits. Yet, this should not be a reason not to employ individuals with disability. Other notable barriers that may impede the success of co-research endeavors include time commitments for both trainers and participants, garnering interest, arranging transportation, and general ableism that perpetuates societal and systemic obstacles to inclusion for those with intellectual and developmental disabilities (e.g., only asking for parents or caregivers reporting on the individual rather than self-report) [4, 9].

Despite barriers, including individuals with intellectual and developmental disabilities as co-researchers has been shown to be successful for research teams [4, 9, 13]. However, opportunities to replicate this process remain rare. Strnadova et al. (2014) proposed that thinking outside of academic publishing could increase the value and relevance of the work to co-researchers. In addition to academia, there may exist alternative avenues to continue training those with intellectual and developmental disabilities as co-researchers—particularly within pre-existing organizations that directly support those with intellectual and developmental disabilities and already participate in research and evaluation. For example, Special Olympics is an international organization that provides a wide array of programmatic activities promoting physical fitness for adults and children with intellectual disabilities. The organization was founded in 1968 and now serves more than 5.5 million athletes. Special Olympics is active in 190 countries and all 50 US states (Special [11]. Aside from athletic competitions, the Special Olympics Healthy Athletes program provides healthcare and health education to many of its athletes, providing more than 2 million health screenings in multiple locations since 1997. Special Olympics also trains athletes as ‘Health Messengers’ to promote the health of fellow athletes and serve as peer advocates (Special [12]. Given the large scope of programmatic activity as well as training capacities, Special Olympics is an ideal partner to expand co-research into the public health domain.

Co-research is a unique, effective, and feasible strategy to bridge the gap between those with intellectual and developmental disabilities and the research teams who study the health of this population. There are still untapped opportunities to train and include those with intellectual and developmental disabilities as co-researchers, especially within organizations like Special Olympics that already serve a large network of people with intellectual and developmental disabilities. To gauge the success of potential co-research opportunities within Special Olympics, we collaborated with Special Olympics Massachusetts, to develop, adapt, implement, and evaluate a public health co-researcher training program for Special Olympics adult athletes.

## Methods

Using funds from a Boston University School of Public Health Practice Innovation Award, we developed and carried out a six-session co-researcher training program in the fall of 2021. The training program incorporated existing resources and literature with new research components—namely an experiential learning component in which participants could formulate their own research questions. Four athletes with previous experience as Health Messengers were recruited to participate. We conducted semi-structured interviews and group theme discussion sessions to evaluate the program. Participants were active partners in the evaluation and are included as co-authors on this manuscript.

### Material development

The six-session co-researcher training program was partially inspired and borrowed from the READI (Research Engagement and Advocacy for Diverse Individuals) curriculum which was developed by the Ausderau Lab at the University of Wisconsin-Madison (Ausderau & Health Research Engagement Development Team, n.d.) [1]. The READI curriculum consists of four individual modules geared towards those with intellectual and developmental disabilities and covers a myriad of health research proficiencies as well as a module regarding self-advocacy. After direct consultation with the University of Wisconsin team, we decided to use two of the four modules. These portions of the READI curriculum were used as the basis for two of our six sessions. In addition to the READI curriculum, we developed an experiential learning experience where participants would utilize their newly acquired research skills to conduct their own experiment and present results at the sixth and final session. We used recommendations from the Toolkit for Remote Inclusive Research to structure the experiential learning portions of the class (Kramer, n.d) [6]. A description of the learning goals and activities of each session are presented in Table 1.

**Table 1:** Timeline and curriculum for public health Special Olympics co-research training

Session	Learning goals	Exercises and activities	Lecture material
Week 1: 10/02/2021	What is health research?	Icebreakers and introductions Health research survey	READI module 1 workbook
Week 2: 10/16/2021	The research process	1–1 interview practice Draft your own research question	READI module 3 workbook
Week 3: 10/23/2021	Begin research project	Left vs. Right hand data collection Draft research idea and questions for coaches	Survey design and confidentiality powerpoint
Week 4: 11/06/2021	Types of research	Hydration survey development and data collection	What is public health powerpoint
Week 5: 11/13/2021	Data analysis and presentation of results	Analyze survey results	Continued final research project work
Week 6: 11/20/2021	Final session wrap-up	Final presentation Pizza party	

### Recruitment

Potential co-research training participants were recruited from Special Olympics Massachusetts athletes who had previously been trained as a Special Olympics Health Messenger. We aimed to recruit 4–6 participants to build rapport and provide tailored attention. In total, four participants were recruited to participate in the co-researcher training. Participants were all White, female, aged from twenties to forties, and had an intellectual disability (specific diagnoses were not required to be disclosed). Participants were of varying cognitive ability and any individual needs and learning accommodations were surveyed following recruitment using a survey also developed by researchers at the University of Wisconsin-Madison and adapted for our use (Additional file 1). Participants were paid $100 in total for participation in the six training sessions—one $50 prepaid Visa gift card at the initiation of the program, and another at the final session. An additional $25 was rewarded for participating in a post-program interview.

### Logistics

The six sessions of the co-researcher training took place Saturday mornings at the Special Olympics Massachusetts site in Marlborough, MA. Sessions were scheduled for 90Â min every other week to avoid potential burnout or scheduling conflicts. The training was conducted indoors, but with adequate social distancing and utilization of masks to avoid potential transmission of COVID-19. Food was provided for co-research participants, and breaks were planned intermittently. Reminder emails were sent to participants and caregivers the Wednesday prior to each session. Each session was led by a [Boston University] Master of Social Work / Public Health student with additional support from another [Boston University] Public Health Master’s student and a faculty advisor.

### Implementation

Prior to the first session of the co-researcher training, the team consulted with the [Boston University Medical Campus] Institutional Review Board (IRB) regarding the ethics of the program and the proposed experiential learning component that would require the creation of a survey for which the participants would elicit responses. The IRB declined the need for institutional review, stating the participants were in a training, their survey was like conducting a “class project” which does not require IRB approval, and the last step is an evaluation which includes the participants as partners. Although no formal consent process was required, participants still received a document detailing what would happen in the program. This document was in Easy Read format and allowed for participants to ask clarifying questions throughout.

Each session comprised of PowerPoint-presented module content, group and individual exercises, as well as collection of participant feedback regarding the session’s content and presentation. To accompany the content of the modules, each participant was given a binder with a workbook and other printed materials containing exercises to apply some of the learning components, such as data analysis and graph drawing. Some exercises were also completed using two of the provided laptops with guidance from staff.

Following the completion of the modules, the staff and participants embarked on the experiential learning project. This entailed the formulation of a research question and the subsequent development of a survey, which was programmed using Qualtrics. Co-research participants distributed the survey to their intended audience and the data was evaluated as a group. During the sixth and final session, the results of the survey were presented by staff and participants to an audience of family, friends, caregivers, and a Special Olympics representative.

### Evaluation

A brief survey (Additional file 1) was given to participants after each session which included 8 statements and checkboxes indicating “I agree” or “I disagree.” The survey assessed the length of the session, need for additional breaks, speed, interest in addressed topics, and perceived support. Additionally, a follow-up interview (Additional file 1) was conducted with each participant via Zoom two months after the final session. The interview was designed to last 30 min to 1 h in length, and assessed lessons learned, favorite activities, personal reflections, and interest in future co-research opportunities. Participants were compensated for completing the follow-up interview.

## Results

### Recruitment and intake

Of the recruitment goal of 4–6 participants, 4 were identified from the Special Olympics Health Messenger program at Special Olympics Massachusetts]. All four identified potential participants agreed to take part in the co-research program. Participants completed intake interviews which assessed and identified learning styles, accommodations, and any one-on-one needs that would be implemented during the program’s sessions.

### Sessions

To avoid burnout or fatigue during the presentation of the modules, adequate time for socialization as well as restroom and snack breaks were incorporated into the weekly sessions. Exercises—mediated by laptop or workbook—were utilized to apply the material from the curriculum and also prevent long periods of lecturing. Only one absence was recorded for the duration of the program.

### Adaptions

Based on participant feedback, we added an additional walk break and reduced the number of PowerPoint presentations. Due to COVID restrictions and IRB guidance, it was decided that participants would not survey coaches or fellow athletes at a Special Olympics tournament as part of the co-research project. Instead, the co-research participants developed an online-based survey disseminated to Special Olympics coaches using email and social media as recruitment tools rather than in-person.

### Experiential learning

A survey was proposed as the research activity that would comprise the experiential learning components of the co-researcher training. Many of the co-researcher participants were interested in diet and exercise as the potential focus of the proposed survey given their background as trained Special Olympics Health Messengers. A consensus was reached to refine the survey topic to hydration. Co-researchers wanted to know if Special Olympics coaches were aware of how much water athletes should be drinking for adequate hydration. The co-researchers decided what questions to ask and how to word them. The online-based survey was developed, programmed using Qualtrics and sent to coaches via Special Olympics Massachusetts listserv, social media, and flyer dissemination. After responses were collected, data analysis and presentation creation were conducted during the fifth session. Co-researchers created bar charts and tables with the data and findings. During the sixth and final session, co-researchers had the opportunity to present data, visuals, and overall findings to Special Olympics employees, friends, and family, followed by a pizza party celebration.

### Evaluation

Feedback regarding the individual sessions was universally positive, although there was feedback that some sessions were content-heavy or that presenters covered material too quickly. As a result, we added breaks and reduced the number of PowerPoint slides. Feedback from the follow-up interviews included reflections that the program provided opportunities for teamwork, learning new research skills, and meeting new people. One participant stated that one of the most valuable skills learned was independence and that ‘it was fun learning about the data and answers.’ Additionally, all participants enthusiastically recalled details and data regarding the hydration survey and the importance of drinking enough water, with many regarding this activity as the highlight of the training program. Data collection, graph interpretation, and survey creation were also mentioned as skills that were gained. All participants expressed interest in participating in more co-research, and when asked if they would recommend the training to program to a friend with a disability, participants unanimously reported back yes, saying it would allow others to “learn about research,” “meet new people,” and “learn to be a researcher like me.”

### Post-program

After the completion of the program, co-researchers have been involved in research projects at Boston University. One co-researcher is part of a Down syndrome specific co-research team and all co-researchers are co-authors of this manuscript and presented this work at the 2022 American Public Health Association conference.

## Discussion

The results of the co-researching curriculum and training program showcase the value of collaboration with and inclusion of individuals with intellectual and developmental disabilities in research endeavors on both a personal and fundamental level. In addition to co-research training participants reporting positive personal experiences, skill building, and learning opportunities, the training program demonstrated that those with intellectual and developmental disabilities bring unique perspectives to the research process and are well-equipped to handle the tasks and responsibilities of being a member of a research team with appropriate adaptions.

### Strategies for success

Several factors ensured the success of our co-researcher training program. Since our sample of co-researchers were recruited from the Special Olympics Healthy Athletes program, they were already versed in health education and advocacy. Our curriculum was also developed by repurposing and building off pre-existing evidence-based materials to train co-researchers, which streamlined the development and planning period. Facilities as well as recruitment were provided and conducted by Special Olympics Massachusetts, therefore, funding did not need to be acquired for space or technology. Additionally, qualitative evidence suggests that the experiential learning component was fundamental to the program’s success and was particularly rewarding for co-research training participants. This component of the program provided an opportunity for training participants to communicate and investigate their own research interests.

### Challenges and obstacles

As noted, a hurdle to co-research with individuals with intellectual and developmental disability is balancing compensation and social safety net programs, such as Supplemental Security Income, that have asset limitations. Other challenges include the writing of co-research into grants and securing funding for projects that include co-researchers. Additional coordination with institutional review boards may also be necessary to build processes and specific approval pathways for co-research studies to receive ethical approval. Lastly, recruiting co-researchers to contribute to research investigations also requires considering and accommodating additional time and work commitments.

### Opportunities

There are several ways this program could be replicated and expanded, particularly as an addition to pre-existing programmatic work for people with intellectual and developmental disability. For example, Special Olympics already performs research and evaluation regarding the health of those with intellectual and developmental disability and conducts health programming and training, namely within the Healthy Athletes Program. Healthy Athletes is a Special Olympics initiative that provides health services and health education to Special Olympics athletes. To date, they have provided over 1 million health screenings to athletes. Screenings also span multiple disciplines, including dentistry, emotional health, nutrition, and eye health. The Healthy Athletes program, in effect, distinguishes Special Olympics as a healthcare provider for those with intellectual and disabilities. This is particularly critical given that health disparities are well-documented among those with intellectual and developmental disabilities who often receive inadequate or substandard healthcare through traditional means [7, 8]. When considering future co-research endeavors, the Healthy Athletes program, coupled with a Special Olympics framework for research, offers a unique opportunity for population-level impact and the potential to incorporate co-research training into pre-existing programmatic activities. Using Special Olympics as an avenue for co-research may also avoid several barriers discussed, such as grant writing and IRB review. For co-researchers, research trainings will teach and improve skills necessary for the workplace and future adaptations to the program can help translate the skills to full employment.

### Limitations

While feedback was overwhelmingly positive from co-research training participants, the program was not without its limitations. A few reviews from participants indicated that presentations during the sessions may have been heavy in dialogue and content. Lessons could have been better enhanced with the inclusion of videos or additional exercises to avoid content feeling like lengthy lectures, which led to some individuals struggling with comprehension and sustained attention. Expanding the program to more than six sessions could have allowed trainers to address issues of attention span and length of material while also allotting more time for participants to practice presentation skills, which was limited in this case. We also recognize that this training program was able to leverage Special Olympics Massachusetts infrastructure, including facilities, recruitment, and equipment to ensure success. Others interested in conducting co-research training programs may require additional resources to plan and fund necessary materials. Additionally, the pilot nature of this program combined with the small sample of four participants indicates the need for further evaluation to identify a more comprehensive list of benefits and limitations. Lastly, the COVID-19 pandemic interfered with our ability to conduct an in-person data collection event with participants at a Special Olympics soccer tournament, limiting our experiential learning experience to a digital survey. Barring any restrictions or risks, future co-researcher endeavors may be able to explore the benefits and challenges of conducting more hands-on data collection.

### Conclusions

Given the overall success of this training program and the reported positive experiences of the co-researchers themselves, we establish that co-research can provide profound insight to the research priorities of individuals with intellectual and developmental disabilities based on their everyday lived experiences, values, and interests. It would behoove researchers and organizations like Special Olympics alike to make more frequent utilization of co-research to identify additional research priorities that affect individuals with intellectual and developmental disabilities and make the research process more accessible and equitable to this community.

## Supplementary Information

**Additional file 1**. Supplemental materials.

## Data Availability

Materials are presented in Additional file 1.
