# Supplementary material for: Developing and implementing a public health co-research program for Special Olympics athletes
Source: Res Involv Engagem. 2023 Jun 19;9:44. doi: 10.1186/s40900-023-00450-5 (PMC10278300; doi:10.1186/s40900-023-00450-5)
Supplement: Supplementary file 1 — Additional file 1. Supplemental materials. [file 40900_2023_450_MOESM1_ESM.pdf]

Boston University

School of Public Health

Department of

Epidemiology

Program Name: Co-Researcher Training Program

Program Instructor: Anna-Mariya Kirova

- You are being invited to participate in a research training program.
- You are in this program because you heard about it from a Special Olympics staff member and you decided to sign up.
- This training program is being taught by Anna-Mariya.

What is this program about?

- We want to train adults with intellectual disabilities to work on research projects related to health and wellbeing.
- To make a good research study, we need to know what people with disabilities want to learn more about.

- We also want to know about the lived experiences of adults with disabilities.

What will I do in the program?

- In this program you will learn about the following:
  - You will learn about what research is.
  - We will list out everyone's interests and decide what health-related topic we want to learn more about as a group.
  - You will learn how to become a better self-advocate and express your needs to others.
  - You will learn how to be a good active listener when communicating with others and when doing interviews for research purposes.
  - You will learn about different ways of collecting data or information about your research topic.
  - You will learn how to present your group's results in front of an audience.
  - You will learn how to protect other people's privacy.
- We may also ask you to answer some questions about your experience doing the program. You do not have to answer any questions that make you upset.

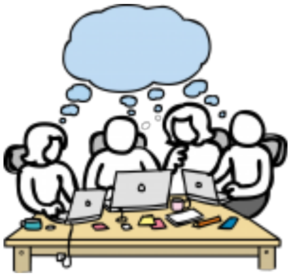

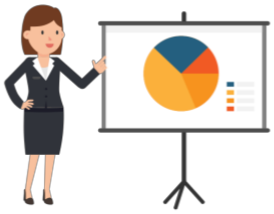

When and where will the program be?

- The program will meet once a week for 6 weeks
- We will meet on __**Saturdays**__ from _**10am**__ to _**12pm**__
- The dates we will be meeting on are: **10/2**, **10/16**, **10/23**, **11/6, 11/7 (Soccer tournament), 11/13, and 11/20**.
- We will be asking parents and coaches health-related questions at the Soccer Tournament on November 7, 2021 at Governor's Academy. **Please plan to attend this additional event even if you are not playing in the tournament.**

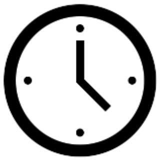

Where will the program meetings be?

- The program meetings will be at the Special Olympics office in Marlborough, MA
- Address**:** 512 Forest St, Marlborough, MA 01752

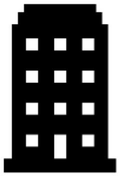

Who will be at the trainings?

- Anna-Mariya will be at every training session.
- There will be 4-6 Special Olympic athletes attending all 6 sessions. You might or might not know some of them already.

- Vicky DiNatale from Special Olympics will be at the trainings to help out Anna-Mariya and the other athletes.
- Special guests may come to some of the sessions to give brief presentations.

Do I have to be in the program?

- If you don’t want to be in this program, you don’t have to.
- No one will be upset if you don’t want to do the program.
- During the program, you don’t have to answer questions that you don’t want to.

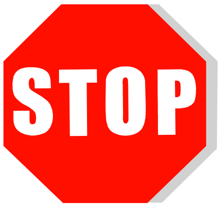

How will this program help me?

- You will learn important skills that you can apply to many different jobs.
- You may find out that you enjoy doing research.
- You can list this program on your resume for future employers to see.
- You will have a chance to meet new people.

Will I be paid to be in the program?

- Yes, you will be receive 2 $50 prepaid Visa gift cards for completing the research training program. You

will get the 1st gift card at the beginning of the program and the 2nd gift card at the last session.

- You will also be reimbursed for transportation to and from the Saturday sessions.

Supports I need For Successful Research Participation

Support<br>☐ I do things on my own<br>☐ I prefer to have a support person come with me<br>☐ I would like to have a research mentor available<br>☐ Other: ___________________<br>**I communicate with:**<br>☐ My voice<br>☐ Sign Language<br>☐ Facial expressions and body language<br>☐ I need an interpreter<br>☐ Assistive device: __________<br>☐ Other: ___________________<br>**To contact me:**<br>☐ An email<br>☐ A phone call<br>☐ Text message<br>☐ Other:____________________<br>**Scheduling**<br>☐ I need ____ weeks notice<br>☐ I schedule myself<br>☐ I need help with scheduling<br>☐ Other: ____________________<br>**Rides for Research Activities**<br>☐ I don't need help with rides<br>☐ I will need a ride<br>☐ I would like help planning rides<br>☐ I need extra time to take the bus<br>☐ I would like to join virtually<br>☐ Other: ______________________	**Meeting length**<br>☐ 30 minutes or less<br>☐ 1 hour or less<br>☐ Up to 2 hours long<br>☐ I need frequent breaks (every ___ min)<br>☐ Other: _____________________________<br>**I Learn Best With:**<br>☐ Talking<br>☐ Video<br>☐ Written Information<br>☐ Other: ___________________<br>**Adapted Versions:**<br>☐ Large font<br>☐ Printed paper handouts<br>☐ Simple language<br>☐ Easy-Read summary (short version)<br>☐ Recorded version<br>☐ Visuals: Pictures instead of words<br>**Paperwork:**<br>☐ I do not need help<br>☐ I would help to **READ**<br>☐ I would like help to **WRITE**<br>**Virtual Meetings and Internet**<br>☐ I have my own device for virtual meetings<br>☐ I need a device to join virtual meetings<br>☐ I have access to the internet<br>☐ I need a hotspot or internet access<br>**Anything else you want to share:**

Supplement 2: Feedback form

Feedback Form

Date: ___ /___ / ___

Directions: Please read each statement. Then place a checkmark in the box if you agree or disagree with the statement.

**Statement**	**I Agree**	**I Disagree**
The sessions were too long	☐	☐
I need more breaks next time	☐	☐
The teacher talked too much or too fast	☐	☐
The information was easy to understand	☐	☐
I liked what I learned today	☐	☐
I am interested in learning more about research	☐	☐
I felt like my voice was heard and others wanted to hear what I had to say	☐	☐
I had enough support to help me do a good job	☐	☐

Thank you for our feedback! Your feedback is important to us.

We will use your feedback when planning future meetings.

Boston University School of Public Health

Rubenstein Lab

SOMA Co-Research Training Interview Guide

Original source: Toolkit for Remote Inclusive Research

Citation: Preparing Individuals with IDD for Engagement in Research During Public Health Emergencies and Disasters Research Team (2020). Toolkit for Remote Inclusive Research: Script for Data Collection. Retrieved January 20, 2022 from http://tamarin-software.com/accessible-toolkit/assets/parts/data-collection/script-for-data-collection.htm

The following interview guide was adapted by Anna-Mariya Kirova, LCSW using key elements from the original source.

Participant Name: ___________________________

Date of Interview: ___________________________

Name of Interviewer(s): _________________________

Interview Steps

1. **Remind participants about their choices.**

Thank you for doing the interview with me. You are going to help our team learn more about your experience doing research. Your ideas will help us learn more about how to support people with disabilities to be researchers.

There are no right or wrong answers. We just want to learn from your ideas and experiences.

If there is a question you do not want to answer, that is OK. You can also ask me to repeat the question or say it in a different way. Please let me know if you want a break. You can take a break at any time.

I might take notes while we are talking to you. The notes are just to help me remember things you say. We will be recording the interview so that we can go back and listen to your answers if we need to. Only the researchers at Dr. Rubenstein’s lab will have access to these recordings.

The interview will be between 1 half hour and 1 hour long.
Do you have any questions?

2. Questions about their experience doing the research training.

I want to learn more about your experiences doing the Boston University research training in the fall of 2021.

- ❑ Why did you want to do research?
- ❑ Why is it important for people with disabilities to do research?
- ❑ What did you learn?
    - Prompt: What are 3 things you learned to do?
- ❑ What is your favorite thing you did?
    - Why did you like it?
- ❑ What was your least favorite thing you did?
    - Why?
- ❑ Would you be comfortable participating in a research team in the future?
    - Why or why not?
- ❑ Would you recommend the training to a friend with a disability?
    - Why or why not?

3. Stories about research

I am going to ask you to tell me some stories about when you were doing research. The stories will help me learn more about your experiences. I will also ask questions after you finish telling the story. These questions will help me learn more about your experience and ideas. I might also ask you to tell stories about other topics.

The topics for the stories are:
1) A time research was important
2) A time research was not very important

Which topic do you want to talk about first?

____

(write the topic they chose)

Go to the page for their topic:

1. A time research was **important-** page **5**
2. A time research was **not very important**-page **6**

A time research was important

Please tell me a story about a time research was important.

- ❑ When was research important?
- ❑ What were you doing when research was important?
- ❑ What helped you do ____?
  (the important activity)
- ❑ How did you learn to do ______________________________________?
  (the important activity)
- ❑ What helped make it important?
- ❑ Did you use any technology, support, or extra information?
  - Prompt: Did anyone help you?
- ❑ How did you feel when you were doing _________________________?
  (the important activity)
- ❑ Who else was there?
- ❑ What was __________________________________ doing?
  (the other person or people)
- ❑ How did you work with __________________________________?
  (the other person or people)
- ❑ How did __________________________________ help the research?
  (the important activity)

Ask what topic they want to talk about next.

A time research was not very important

Please tell me a story about a time research was not very important.

- ❑ When was research not very important?
- ❑ What were you doing when research was not important?
- ❑ Did you finish doing ______________________________________? (the activity)
- ❑ Did you learn something from doing _________________________? (the activity)
- ❑ What made it not important?
- ❑ Did you use any technology, support, or extra information?
    - o Prompt: Did anyone help you?
- ❑ How did you feel when you were doing _______________________? (the boring activity)
- ❑ Who else was there?
- ❑ What was ________________________________ doing? (the other person or people)
- ❑ How did you work with ________________________________? (the other person or people)
- ❑ How would you change ___________________ to make it important? (the activity)

Ask what topic they want to talk about next.

4. Closing statement

Thank you very much for you doing the interview. I enjoyed talking with you. If you have any questions, you can contact me. If you decide you do not want us to include anything you told us, you can let us know. It is ok to change your mind.

Is there anything you told us that you do not want us to include when we write about the research? We will not say your name.

Check off their response

- ❑ Yes, please do not include ________________________________
- ❑ No, you can include everything

I might have questions about what you told me. If we talk again, we will talk for a shorter amount of time. The second conversation will probably be about 15 minutes. If I have questions, is it ok to contact you again?

Check off their response

- ❑ Yes, it is ok to contact me again.
- ❑ No, please do not contact me again.

Thank you so much! It was fun to talk with you and you helped us a lot.
